# Health Seeking Behaviours and Knowledge of Infectious Disease Risks in Western Australian Travellers to Southeast Asian Destinations: An Airport Survey

**DOI:** 10.3390/tropicalmed1010003

**Published:** 2016-07-18

**Authors:** Chloe A. Thomson, Robyn A. Gibbs, Carolien Giele, Martin J. Firth, Paul V. Effler

**Affiliations:** 1 The University of Western Australia, 35 Stirling Highway, Perth 6009, Australia; Martin.Firth@uwa.edu.au (M.J.F.); Paul.Effler@health.wa.gov.au (P.V.E.); 2 Communicable Disease Control Directorate, Western Australia Department of Health, 227 Stubbs Terrace, Perth 6008, Australia; Robyn.Gibbs@health.wa.gov.au (R.A.G.); Carolien.Giele@health.wa.gov.au (C.G.)

**Keywords:** travel health, travel health advice, disease prevention, communicable disease

## Abstract

As the number of Australians engaging in short-term international travel increases, so does the opportunity for importing overseas-acquired infectious diseases. This study aimed to determine knowledge of infectious disease risks and pre-travel health advice (PTHA) seeking behaviour among Western Australians travelling to Bali, Indonesia or Thailand. Passengers departing from Perth International Airport were invited to participate in a self-administered survey. The survey determined PTHA seeking behaviour, knowledge of specific disease risks, and expected disease-prevention behaviours abroad. Multivariate regression modelling was used to assess demographic and travel-related factors associated with seeking PTHA. Responses from 1334 travellers were analysed. The proportion correctly identifying specific overseas disease risks ranged from 27% to 98%. High levels of planned disease-preventive behaviours were reported; however only 32% of respondents sought PTHA for their trip, most commonly from friends/family (15%) or a GP (14%). Many travellers (87%) made online travel purchases, but few (8%) used the Internet to source PTHA. WA travellers to Bali and Thailand were unlikely to seek PTHA and knowledge varied regarding infectious disease risks associated with travel. High rates of internet use when planning travel may provide an opportunity for destination-specific health promotion messaging and should be explored.

## 1. Introduction

Over the past decade, the number of Australian residents departing on short-term international trips has risen two-fold, with over 9,110,000 departures in 2014 [1]. As the number of Australians engaging in international travel increases, so does the opportunity for importing overseas-acquired infectious diseases into Australia [2].

**Figure 1:**
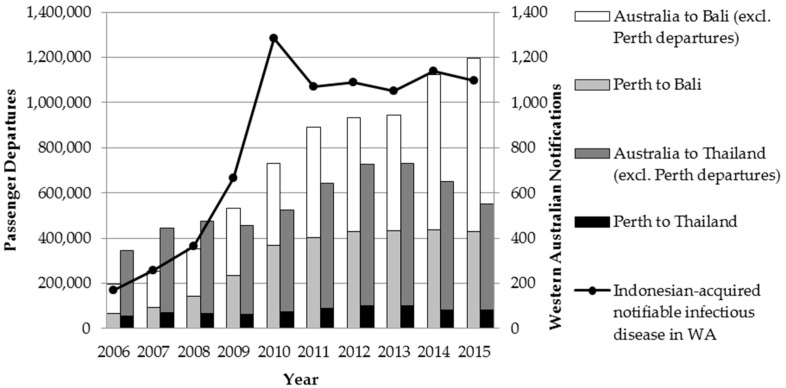
Airline passenger departures to Bali, Indonesia and Thailand, and communicable disease notifications in Western Australia attributed to travel to Indonesia, from January 2006–December 2015.

Bali, Indonesia is a popular destination for Australian travellers, particularly travellers from Western Australia (WA). In 2014, there were more than 430,000 departures from Perth to Bali, an increase of more than six-fold since 2006 [1,3] (Figure 1). Passenger numbers to Thailand have remained relatively constant but substantial, with over 80,000 passenger departures from Perth to Thailand in 2014 [3].

Increased travel to Bali, Indonesia has resulted in an increase of Indonesian-acquired infectious diseases amongst travellers returning to WA. From 2006 to 2012, the proportion of all overseas-acquired infections in WA attributed to Indonesia rose from 10% to 29% [4]. The two notifiable diseases with the highest number of cases acquired in Indonesia are dengue fever and salmonellosis; from 2006 to 2012 Indonesian-acquired dengue fever notifications in WA rose 46-fold, and Indonesian-acquired salmonellosis notifications in WA increased nine-fold [4]. In addition, the number of post-exposure prophylaxis (PEP) treatments for potential rabies exposures in Bali is increasing, largely due to monkey exposures [5,6]. Australia has eliminated endemic measles transmission, so an increased risk of exposure to circulating measles travellers while overseas is also a concern [7]; in 2013, six WA travellers returning from Indonesia developed measles, accounting for 43% of all measles diagnoses in WA that year [8]. Given increasing numbers of dengue, salmonellosis and measles infections acquired overseas, developing effective risk reduction strategies for travellers is a priority [9,10,11]. Determining travellers’ knowledge of infectious disease risks, current practices with regard to seeking pre-travel health advice (PTHA), and their intent to engage in risk reduction behaviours while travelling is an important step in developing targeted disease prevention messages. Prior travel health surveys have usually focused on tourists from Europe and North America [11,12,13,14,15,16,17,18,19] and findings from these studies may not be applicable to Australian travellers because of differences in access to health care, culture, and travel health literacy [10,20].

The aims of this study were to assess the knowledge of WA travellers with regard to infectious disease risks present in Bali, their intent to engage in risk reduction behaviours, and to determine the proportion who sought PTHA. Travellers to Thailand were also surveyed and their responses were compared to those from the Bali travellers to determine if there were important differences between Bali travellers and those going to another popular destination in Asia.

## 2. Materials and Methods 

### 2.1. Participant Selection and Exclusion

Participants were selected while walking en route to emigration at Perth International Airport. To be eligible for this study, participants had to be: WA residents aged 18 years or older travelling to Bali, Indonesia or Thailand (any region); staying at the destination for at least one night; and, able to read and give verbal consent in English. Participation was limited to one person per household travelling together.

### 2.2. Questionnaire 

Potential participants were asked to complete a self-administered questionnaire designed to take less than 10 min to complete. The questionnaire collected basic demographic information, specifics regarding travel plans, details of any PTHA sought, and intended behaviours to reduce the risk of acquiring an infectious illness. Respondents were also assessed regarding their knowledge of dengue, salmonellosis, rabies and measles, and the risk of potential exposure while abroad. Prior to data collection at the airport, the survey was distributed to 20 individuals, who completed the survey independently. Minor edits were made to the survey as a result.

### 2.3. Questionnaire Administration

Twenty-eight flights from Perth International Airport to Bali and ten to Thailand departing over a two-week period during May 2014 were selected. A uniformed representative of the WA Department of Health (DOH) approached a convenience sample of passengers waiting to pass through airport emigration, enquiring if they would participate. The WA DOH representative remained nearby to clarify any questions and collect the survey after completion.

### 2.4. Data Analysis

The proportion of travellers who sought PTHA, the source of this travel advice, the proportion who were aware of specific health risks, and the proportion of travellers planning to take preventive action to mitigate these risks was calculated. Proportions were compared for different demographic groups, and destinations, using the Pearson’s chi-squared test. Analyses were conducted using EpInfo v.7.1.4 (Centres for Disease Control and Prevention, Atlanta, GA, USA) and Stata Statistical Software v.14 (StataCorp LP, College Station, TX, USA).

Demographic and other characteristics significantly associated with seeking PTHA in univariate analyses (*p* ≤ 0.1) were included in a multivariate logistic regression model, with PTHA (yes/no) as the dependent variable. Backwards stepwise elimination was performed to establish independent predictors for seeking PTHA among travellers; a *p*-value of ≤0.05 was considered significant in the regression analysis.

This research was approved by the Department of Health WA Human Research Ethics Committee (reference number 2014/19) and The University of Western Australia Human Research Ethics Committee (reference number RA/4/1/6820).

## 3. Results

A total of 1560 travellers was approached to participate, of which 102 (7%) refused. After excluding passengers who reported residing outside WA (*n* = 61) and surveys that were completed incorrectly (*n* = 63), responses from 1334 travellers were available for analysis; this included 1091 passengers destined for Bali and 243 to Thailand.

### 3.1. Traveller Demographics

Most respondents identified themselves as tourists (90%), with small numbers travelling for business (3%) or to visit family and friends (4%) (Table 1). Almost 60% were travelling with another member of their household, and 15% of all respondents were travelling with children. The mean duration of stay for all travellers was 8.7 days (SD = 9) and the mean age of respondents was 42 years (SD = 14). The location of accommodation at the destination was clustered around the main tourist hubs, with 80% of travellers to Bali staying in Kuta and surrounding areas (Legian and Seminyak), and 64% of travellers to Thailand staying in Phuket or Bangkok. There were slightly more female respondents (53%) than male respondents (47%).

### 3.2. Pre-Travel Health Advice 

Thirty-two percent of travellers reported seeking PTHA for the current trip and 58% had sought PTHA for a previous overseas trip to any destination. The most popular sources of PTHA were friends and family (15%) and general practitioners (GP) (14%) (Table 2). Whilst 8% of travellers sought health advice online, only 4% visited the official Australian government travel advice website [21]. Half of all respondents (50%) reported not seeking PTHA because they travel frequently and felt they were aware of the risks, while 10% did not seek PTHA because they were in good health, so they felt it was not necessary. A further 7% did not seek PTHA because they believed that there were little or no health risks at their destination. 

Significant, independent predictors of seeking PTHA included travelling with children (OR = 2.01; 95%CI = 1.46–2.76; *p* ≤ 0.001), staying in hotel accommodation (OR = 1.56; 95%CI = 1.15–2.10; *p* = 0.004), and female gender (OR = 1.35; 95%CI = 1.07–1.71; *p* = 0.013). Travellers who had obtained PTHA in the past were twice as likely to seek advice for this trip (OR = 2.13; 95%CI = 1.66–2.73; *p* ≤0.001), whilst travellers who had purchased flights or accommodation directly through an airline or accommodation website were less likely to have sought PTHA (OR = 0.72; 95%CI = 0.57–0.92; *p* = 0.009). The proportion of travellers that sought PTHA was similar between those destined for Bali (32%) or Thailand (36%) and intended destination was not a significant predictor of seeking PTHA in the multivariate model.

### 3.3. Health Risk Preventive Behaviours

A majority (85%) of travellers purchased travel insurance for this trip (Table 3). Travellers also reported a high rate of planned preventive measures; 82% planned to use insect repellent and 70% to use alcohol-based hand sanitiser. Conversely, only 16% and 25% of travellers planned to use mosquito nets and mosquito protective clothing, respectively. One quarter (25%) of travellers had reviewed their vaccinations before travelling and 26% reported that they had not been vaccinated against measles or were unsure of their vaccination status. 

### 3.4. Health Risk Knowledge

The largest gap in travellers’ health risk knowledge concerned the heightened risk of exposure to measles in Bali/Thailand (Table 4). Only 27% of travellers identified that the risk of acquiring measles in Bali/Thailand was greater than that in Australia, despite Australia being declared measles-free [7]. Knowledge regarding the risk of mosquito-borne illness was high, with over 80% of respondents agreeing that day-biting mosquitoes can transmit serious disease and insect repellent should be used when outside in Bali/Thailand. Knowledge of foodborne disease risks varied. Only 38% of travellers identified that ice at their destination was a potential source of illness and just over half (52%) identified salads as a potential risk. Knowledge scores were highest regarding the potential risk of rabies with over 90% correctly identifying that urgent medical attention was required after a bite from a monkey or dog in Bali/Thailand; still, one in 10 travellers (*n* = 149) thought it was safe to interact ‘feed and play with’ monkeys at tourist venues in Bali/Thailand.

## 4. Discussion

This study found that only about a third of travellers sought any health advice prior to travelling to Bali or Thailand. This figure is below the 52% of Australians reported in a 2010 airport survey [10] and levels reported in previous airport surveys from other countries [9,11,16,22,23,24]. In addition, in our setting, the proportion of travellers who consulted a professional source for pre-travel health advice was lower than in other studies, with just one in five consulting a GP, travel doctor, pharmacist, travel agent or government website before departing. Higher rates of travellers seeking professional advice have been reported from studies in Sydney (36%), Spain (60%) and the United States of America (52%) [10,13,19]. 

Over half of the respondents in this study indicated they felt that PTHA was not necessary because they travelled to their destination frequently and were familiar with the potential health threats. This response contrasts with other airport surveys of travellers to Southeast Asia, where concern about vaccinationside-effects, and a perceived low-risk destination were the most common barriers to seeking PTHA [24,25]. Whilst frequent travellers may be better informed concerning the more high profile risks (e.g., rabies), it is unlikely to be true for new or evolving disease risks, such as a measles outbreak. Travellers who rely on non-professional sources of PTHA (e.g., family and/or friends, travel discussion forums on the internet) may not receive the most current information. Compared with a previous Canadian study, in which 80% of travellers correctly identified both salad and ice as a potential health risk [17], knowledge of the risk posed by these items was lower for Western Australian travellers, with 52% and 49% correctly identifying ice and salad, respectively, as potential risks. More hotels and restaurants in Southeast Asia are using filtered water when preparing ice for beverages; however, this move is by no means universal. Whilst many food and water-borne illnesses in Southeast Asia are not life threatening, they can incur economic costs, including working days lost to illness.

Travellers’ knowledge was highest regarding the potential exposure to rabies in Asia. Considering the financial, time, and emotional costs of rabies post-exposure prophylaxis, it is important that all travellers are aware of the risk of rabies exposure, especially those planning to interact with mammalian wildlife.

### 4.1. Limitations

There are limitations to this study. As with most cross-sectional studies, this study only captured the pre-travel health seeking behaviours of travellers over a limited (two-week) period. The travellers during this period may not have been reflective of travellers throughout the year. Recruitment of participants was subject to selection bias. Those with strong health concerns may have been more inclined to participate in the study (self-selection), just as those with a more casual attitude may have declined to participate. Travellers who are nervous flyers/travellers may have also declined to participate. The survey tool developed within with WA DOH was not validated before use. As with most surveys, completion of the survey was also subject to recall bias, and although planned health risk prevention behaviours were recorded, we could not assess if the traveller actually engaged in these behaviours.

### 4.2. Strategies for Promoting Risk Reduction Behaviours 

This survey demonstrated gaps in health knowledge and low levels of seeking pre-travel health advice among travellers to Bali and Thailand. Changing PTHA-seeking behaviours among travellers is difficult, especially when many of the travellers claim to already be aware of any potential health risks. One potential opportunity for increasing delivery of health promotion messages might be to target travellers through the internet. In our cohort, 87% of travellers bought either their airline tickets or their accommodation online however only 8% used the internet to access PTHA. The Australian Department of Foreign Affairs and Trade coordinates the smartraveller website [21], which provides readily-available, up-to-date travel advice. Strategies should be developed to direct internet travel traffic to sources such as this. The 85% of travellers purchasing travel insurance suggests a further opportunity to reach travellers on the internet before they travel. Securing advertising space with links to smarttraveller.gov.au on travel insurance or travel flight/accommodation websites is also an opportunity to be explored. In particular, major airline and hotel websites should be targeted, to capture those travellers using those sites and who may otherwise be less likely to access PTHA. This could obviate the need for the traveller to have to actively search for PTHA and hopefully result in greater exposure to quality health promotion messages as travellers make their usual pre-travel purchases.

## 5. Conclusions 

These data build upon limited information on the knowledge and behaviours of Australian travellers with regard to the risk of acquiring an infectious disease while abroad. In our study, travellers were unlikely to seek health advice prior to departure, and when they did, friends and family members were the most common sources of information. Gaps in knowledge regarding the risk of foodborne illness and measles exposure were evident. Given that most travellers reported using the internet to make their travel arrangements, innovative web-based approaches for providing accurate, current, pre-travel health advice should be developed and evaluated.

## Figures and Tables

**Table 1:** Traveller demographics for Western Australians travelling to Bali (Indonesia) and Thailand.

Characteristics	Number of Travellers	% (*n* = 1334)
Destination		
Bali	1091	82
Thailand	243	18
Mean age (SD)	42 (14)	
Gender ^a^		
Male	624	47
Female	709	53
Country of birth		
Australia	913	68
Other	420	32
Purchased flights/accommodation ^b^		
Travel agent ^a^	248	19
Internet, directly from airline or accommodation provider ^a^	640	48
Internet, third party travel website	462	35
Other ^a^	60	5
Education		
Some high school	133	10
Completed high school	335	25
Technical certificate	472	35
University	276	21
Postgraduate	114	9
Purpose of travel/visit		
Holiday/tourist	1208	90
Business	42	3
Visiting friends/family	54	4
Work	10	1
Other	20	2
Travel companions		
Yes	797	60
No	537	40
Adults in the same household ^a^	789	59
Children in same household ^a^	197	15
Duration of stay (days)		
Mean (SD)	8.7 (9)	
Accommodation ^a^		
Hotel	1028	77
Hostel	13	1
Private villa	229	17
Staying with local family/friend	47	4
Unsure/unspecified	13	1
PTHA sought previously ^a^		
Yes	772	58
No	562	42

**^a^** Variables with a univariate *p*-value ≤ 0.1 in cross tabulation with PTHA were included as independent variables in a binary logistic regression. **^b^** Participant could choose multiple answers.

**Table 2:** Features of pre-travel health advice sought by Western Australians travelling to Bali (Indonesia) and Thailand.

	Number of Travellers	% (*n* = 1334)
**PTHA sought before this trip:**		
Yes	430	32
No	904	68
**Source of advice ^a^**		
General practitioner	188	14
Pharmacist	94	7
Government website	48	4
Travel agent	46	3
Travel doctor	25	2
**Any professional advice**	300	22
Friends or family	201	15
Internet	112	8
Travel guide	33	3
**Reason for not seeking PTHA ^a^**		
Travel frequently to the destination	669	50
Traveller is in good health	130	10
There is no risk	99	7
There was no time	45	3
Traveller didn’t know where to look	33	2
Cost	33	2

**^a^** Participant could choose multiple answers.

**Table 3:** Risk preventive behaviours reported by Western Australians travelling to Bali (Indonesia) and Thailand.

	Number of Travellers	% (*n* = 1334)
**Risk prevention behaviour:**		
**Insect repellent use**		
Yes	1090	82
No/don’t know/blank	244	18
**Alcohol hand sanitiser use:**		
Yes	933	70
No/don’t know/blank	401	30
**Mosquito net use:**		
Yes	207	16
No/don’t know/blank	1127	84
**Mosquito protective clothing use**		
Yes	332	25
No/don’t know/blank	1002	75
**Reviewed vaccination history**		
Yes	333	25
No/don’t know/blank	1001	75
**Measles immunity (vaccination or previous infection)**		
Yes	1163	87
No/don’t know/blank	171	13
**Purchased travel insurance**		
Yes	1137	85
No/don’t know/blank	197	15

**Table 4:** Health risk perception reported by Western Australians travelling to Bali (Indonesia) and Thailand.

	Number of Travellers	% (*n* = 1334)
**Health risk knowledge**		
**The risk of someone catching measles in Bali/Thailand is about the same as catching it in Australia**		
Correct (disagree)	364	27
Incorrect/don’t know/blank	970	73
**Mosquitoes that bite in the daytime are a nuisance in Bali/Thailand, but they don’t transmit serious diseases**		
Correct (agree)	1096	82
Incorrect/don’t know/blank	238	18
**It is important to always use mosquito repellent when outdoors in Bali/Thailand**		
Correct (agree)	1148	86
Incorrect/don’t know/blank	186	14
**The ice from the bigger hotels in Bali/Thailand is generally safe to serve in drinks**		
Correct (disagree)	654	49
Incorrect/don’t know/blank	680	51
**Salads (ie uncooked fruit and vegetables) are safe to eat in Bali/Thailand**		
Correct (disagree)	691	52
Incorrect/don’t know/blank	638	48
**Any eggs eaten in Bali/Thailand should be cooked thoroughly (not runny) and served hot**		
Correct (agree)	863	65
Incorrect/don’t know/blank	471	35
**You should seek urgent medical attention if you are bitten by a dog or other mammal in Bali/Thailand**		
Correct (agree)	1305	98
Incorrect/don’t know/blank	29	2
**It is safe to feed and play with monkeys at tourist venues in Bali/Thailand**		
Correct (disagree)	1182	89
Incorrect/don’t know/blank	152	11
**You should be treated to prevent rabies if you are scratched or bitten by a monkey in Bali/Thailand**		
Correct (agree)	1251	94
Incorrect/don’t know/blank	83	6
